# Plasma Metabolome Mediates the Link Between Chronic Pain and Brain Disorders: A Large‐Scale Cohort and Genetic Analysis

**DOI:** 10.1002/cns.71234

**Published:** 2026-11-10

**Authors:** Lin Pan, Wenzhuo Yang, Zijian Chen, Leyan Chen, Kaijun Sun, Yuxin Liu, Weijie Ding, Xinhui Wang, Hongyu Chen, Yonggao Mou, Yinsheng Chen, Sheng Zhong

**Affiliations:** ^1^ Department of Neurosurgery, State Key Laboratory of Oncology in South China, Guangdong Provincial Clinical Research Center for Cancer Sun Yat‐sen University Cancer Center Guangzhou China; ^2^ Department of Neurology, Neuroscience Research Center The First Hospital of Jilin University Changchun China; ^3^ Department of Oncology First People's Hospital of Xinxiang & The Fifth Affiliated Hospital of Xinxiang Medical College Xinxiang China

**Keywords:** chronic pain, inflammatory index, metabolic signature, neurological disorders, psychiatric disorders

## Abstract

**Background:**

Chronic pain has been linked to neurological and psychiatric disorders, yet the metabolic mechanisms driving this association remain unclear.

**Methods:**

Leveraging data from 174,630 UK Biobank participants, we examined associations of 251 plasma metabolites with chronic pain across seven body sites and constructed a metabolic signature using elastic net regression. Cox proportional hazards models were applied to explore the associations of chronic pain and the metabolic signature with 10 incident brain disorders. For outcomes with significant associations, we evaluated genetic causality of the identified metabolites using Mendelian randomization (MR). Mediation analyses were conducted to evaluate inflammatory pathways involving eight markers, such as platelet count and C‐reactive protein.

**Results:**

Most metabolites were significantly associated with chronic pain (FDR‐corrected *p* < 0.05), with 88 metabolites forming a metabolic signature. During a median follow‐up of 15.6 years, an increasing number of chronic pain sites and a higher metabolic signature were associated with increased risks of depression, anxiety, and ischemic stroke (IS). Systemic inflammation, particularly platelet and leukocyte counts, partially mediated the effect of the metabolic signature on depression (4.33%) and IS (9.52%). Furthermore, MR analyses suggested potential causal roles for several metabolites in these outcomes, such as docosahexaenoic acid (DHA).

**Conclusion:**

Our findings highlight the pivotal role of metabolic dysregulation in the connection between chronic pain and brain disorders. These results offer valuable insights into inflammation‐mediated mechanisms and identify potential metabolic biomarkers for prevention.

## Graphical abstract

This study identified the metabolic signature of 88 metabolites associated with chronic pain and increased risks of depression, anxiety, and ischemic stroke. We demonstrated that systemic inflammation (specifically platelet and leukocyte counts) acts as a potential driver linking this signature to disease risk. Furthermore, we identified specific metabolites with causal roles in these conditions, providing viable biomarkers for disease intervention. Created with BioRender.com.

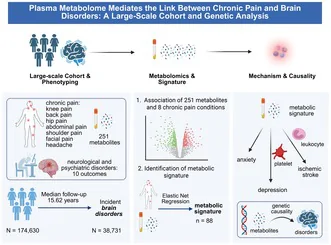

## 1 Introduction

With the global population growth and aging, the rising prevalence of neurological and psychiatric disorders has garnered increasing attention. Among these disorders, stroke is the second leading cause of death worldwide, accounting for approximately 6.5 million deaths annually [1]. Depression remains a leading cause of disability [2]. Given their high prevalence and irreversibility, strengthening primary prevention to preserve brain health is an urgent public health priority.

Chronic pain, typically defined as pain persisting for more than 3 months, is a heterogeneous and multifactorial condition shaped by environmental, biopsychosocial, and genetic influences [3, 4]. Accumulating observational studies suggest a detrimental linkage between chronic pain and the development of neurological and psychiatric disorders. For instance, individuals with chronic pain have been shown to have a significantly higher prevalence of depression [5]. Additionally, recent studies have reported shared genetic architecture between chronic pain and psychiatric traits [6, 7, 8, 9, 10]. Significant genetic correlations have been observed between pain phenotypes and diseases such as anxiety and substance use disorders. These findings suggest that a common genetic susceptibility underlies comorbidity between chronic pain and psychiatric disorders. However, prior studies are limited by small samples, insufficient confounder adjustment, and lack of data on the incidence of specific brain health outcomes. There is an urgent need to evaluate pain status and clarify its relationships with brain disorders.

High‐throughput metabolomics enables comprehensive profiling of small‐molecule metabolites during specific physiological states, capturing the consequences of genetic and environmental influences [11]. Prior studies indicate that chronic pain can substantially affect multiple metabolic pathways [12]. However, existing research has mainly examined associations between chronic pain and individual metabolites, while overall chronic pain‐related metabolic signatures remain insufficiently characterized. Abnormal levels of circulating metabolites have also been linked to the risk of brain diseases. For instance, cholesterol levels in medium HDL correlate with a reduced risk of stroke, while increased acetate levels are associated with dementia [13, 14]. Despite these findings, the extent to which metabolic alterations may link chronic pain with incident neurological or psychiatric outcomes remains unclear. Moreover, plasma metabolites may influence nervous system function through inflammation, oxidative stress, and disruptions in energy metabolism [15]. Inflammatory factors have also been implicated in depression, anxiety, and many neurological disorders [16]. However, the interplay between metabolic signature and inflammation in brain disorders remains to be investigated.

Overall, by integrating metabolomics into chronic pain research and elucidating inflammation‐related mechanisms, this study could provide novel insights into the prevention of brain disorders.

## 2 Methods

### 2.1 Study Population

The UK Biobank (UKB) project is a large population‐based prospective cohort study that enrolled over 500,000 participants during 2006–2010. Details are described elsewhere [17]. This study was conducted under UKB application number 217841.

In the present study, participants were excluded if they withdrew from UKB, had brain disorders at baseline, or lacked pain questionnaire or NMR metabolomics data. Finally, 174,630 participants were eligible for this study. A flowchart illustrating the selection criteria is provided in Figure S1.

### 2.2 Pain and Metabolite Profiling Assessment

At baseline, participants reported the types of pain they experienced in the last month via a touchscreen questionnaire. Participants could select from seven body regions (knee, hip, abdomen/stomach, back, shoulder/neck, face, head), a whole‐body pain option (general pain), or “none of the above”. Multiple selections were permitted for the seven regional sites, whereas general pain allowed only a single choice. Individuals reporting pain at any site or “all over the body” were then asked whether it had persisted for more than 3 months, which was classified as chronic pain. In line with prior studies [6, 8], multisite chronic pain was defined as the sum of body sites with chronic pain across the seven regional sites, ranging from 0 to 7. This measure was used as a proxy for the extent of chronic pain (Table S1).

NMR metabolomic profiling data were generated using the Nightingale Health platform [18]. These phases encompassed 251 metabolic biomarkers, measured in EDTA plasma from approximately 280,000 UK Biobank participants (Table S2).

### 2.3 Neurological and Psychiatric Disorders Events

The primary outcome was defined as a composite of first‐episode neurological and psychiatric disorders, including ischemic stroke (IS), intracerebral hemorrhage (ICH), subarachnoid hemorrhage (SAH), depression, anxiety, bipolar disorder (BD), schizophrenia (SCZ), mania, Alzheimer's disease (AD), and epilepsy. These diseases were identified using self‐reports, hospital inpatient records, death registry, and “first occurrence of health outcomes” category.

### 2.4 Assessment of Inflammation Index

To examine whether inflammation mediates the association of the metabolic signature with brain disorders, we selected immune parameters implicated in distinct inflammatory pathways: absolute counts of lymphocytes, leukocytes (white blood cells), platelets, and neutrophils; C‐reactive protein (CRP); the neutrophil‐to‐lymphocyte ratio (NLR) and platelet‐to‐lymphocyte ratio (PLR); and the systemic immune‐inflammation index (SII). SII was computed as platelet count × neutrophil count/lymphocyte count [19].

### 2.5 Assessment of Covariates

A total of 11 covariates associated with brain disorders were considered in the analyses, including age, sex, ethnicity, educational attainment, the Townsend deprivation index, smoking status, alcohol consumption, weight status based on body mass index, family history of brain disorders, and prevalent hypertension and diabetes.

### 2.6 Identification of Chronic Pain Condition‐Related Metabolites

Multivariable linear regression models were used to assess the associations between 251 metabolite levels and pain conditions across seven body sites, as well as the number of chronic pain sites. To control for potential confounders, these models were adjusted for all 11 aforementioned covariates. Prior to analysis, we applied log transformation and standardized all metabolites to the same scale. Significance was defined by a false discovery rate (FDR)‐corrected *p* < 0.05.

To derive a metabolic signature for chronic pain, we fitted an elastic net regression, which blends lasso and ridge regression penalties to address multicollinearity, reduce overfitting, and enable feature selection [20]. The multisite chronic pain was regressed on all 251 standardized metabolites, and the optimal penalty parameter λ was selected via 10‐fold cross‐validation using the one‐standard‐error rule. We then computed a total metabolic signature as the weighted sum of the selected metabolites, with weights equal to the elastic net coefficients.

### 2.7 Associations of Pain Conditions and Metabolic Signature With Neurological and Psychiatric Disorders

In the UKB prospective cohort, we estimated HRs and 95% CIs using Cox proportional hazards models. We conducted two analyses: (1) associations of the number of chronic pain sites and each individual pain site with the brain disorders; and (2) associations of the metabolic signature with the brain disorders. Multiple testing correction was applied to each model, and statistical significance was defined as a Bonferroni‐corrected *p* < 0.05. Kaplan–Meier curves were generated to compare risk across metabolic signature groups and categories of chronic pain sites.

We also assessed the association between the number of chronic pain sites and prevalent neurological or psychiatric disorders among 146,460 participants, after excluding individuals with missing data on chronic pain, NMR metabolomics, or covariates, as well as those who developed incident brain disorders during follow‐up (Figure S1). First, logistic regression models were fitted to estimate the odds ratios (ORs) and 95% CIs for each prevalent disease in relation to the number of chronic pain sites, adjusting for all covariates. Additionally, we characterized the comorbidity of chronic pain with neurological and psychiatric disorders among these 146,460 participants. Participants were classified based on the number of prevalent neurological or psychiatric disorders across the 10 predefined outcomes, using ordered categories of 0, 1, or ≥ 2 disorders. To evaluate the specific composition of prevalent conditions, we independently categorized participants by disorder type: no neurological or psychiatric disorder, neurological disorder only, psychiatric disorder only, or both neurological and psychiatric disorders. Ordinal logistic regression was employed to examine the associations of the number of chronic pain sites and site‐specific chronic pain with prevalent neuropsychiatric comorbidity, adjusting for covariates. To account for multiple comparisons across the prevalent outcomes, significance was defined by a Bonferroni‐corrected *p* < 0.05.

### 2.8 Subgroups and Sensitivity Analysis

We assessed heterogeneity via stratified analyses. To evaluate robustness, we performed two sensitivity analyses: (1) excluding participants who developed incident outcomes within the first 3 years of follow‐up; and (2) additionally adjusting for antidepressants, anti‐anxiety medications, lipid‐lowering medications, and aspirin use.

### 2.9 Causal Inferences Using MR

We conducted MR analyses to infer causal effects of genetically predicted metabolic profiles on the risks of brain disorders. For all identified metabolites, summary statistics were sourced from a previously published study of 115,078 UKB participants of European ancestry. GWAS summary data for depression were obtained from FinnGen. The summary statistics for anxiety were obtained from a recent study [21]. The data for IS were retrieved from GWAS Catalog [22]. We selected significant SNPs (*p* < 5 × 10^−8^) and performed LD clumping (*r* ^2^ < 0.1; 10,000 kb). The inverse‐variance weighted (IVW) method was the primary estimator [23]. MR‐Egger, weighted median, simple mode, and weighted mode were implemented as sensitivity approaches. Heterogeneity was evaluated with Cochran's *Q* test, and horizontal pleiotropy was assessed using the MR‐Egger intercept [24]. To account for multiple testing, FDR correction was applied. Associations with FDR‐corrected *p* < 0.05 were considered statistically significant, and a nominal *p* < 0.05 was considered suggestive.

### 2.10 Mediation Analyses

For the significant associations identified in the Cox models, we performed mediation analyses to evaluate two potential pathways. First, we implemented a standard three‐variable path model to quantify the extent to which the metabolic signature mediated the associations between multisite chronic pain and incident depression, anxiety, and IS. Second, we assessed whether inflammatory indexes mediated the associations between the metabolic signature and incident depression, anxiety, and IS. All analyses were performed using the mediation package in R.

Detailed descriptions of all the above‐mentioned method sections are provided in the Supplementary Methods (Data S1).

## 3 Results

### 3.1 Participants' Characteristics

Figure 1 (A) presents the overall study design. Of the 174,630 included participants, the mean (SD) age was 56.56 (8.0) years, and 54.19% were male (Table 1). The number of chronic pain sites ranged from 0 to 7. Across increasing chronic pain sites, participants were more socioeconomically deprived, more often non‐white, more likely to be current smokers and nondrinkers, had lower educational attainment, slightly higher BMI, a higher prevalence of diabetes and hypertension, and a notably higher usage of antidepressants and aspirin.

**FIGURE 1:**
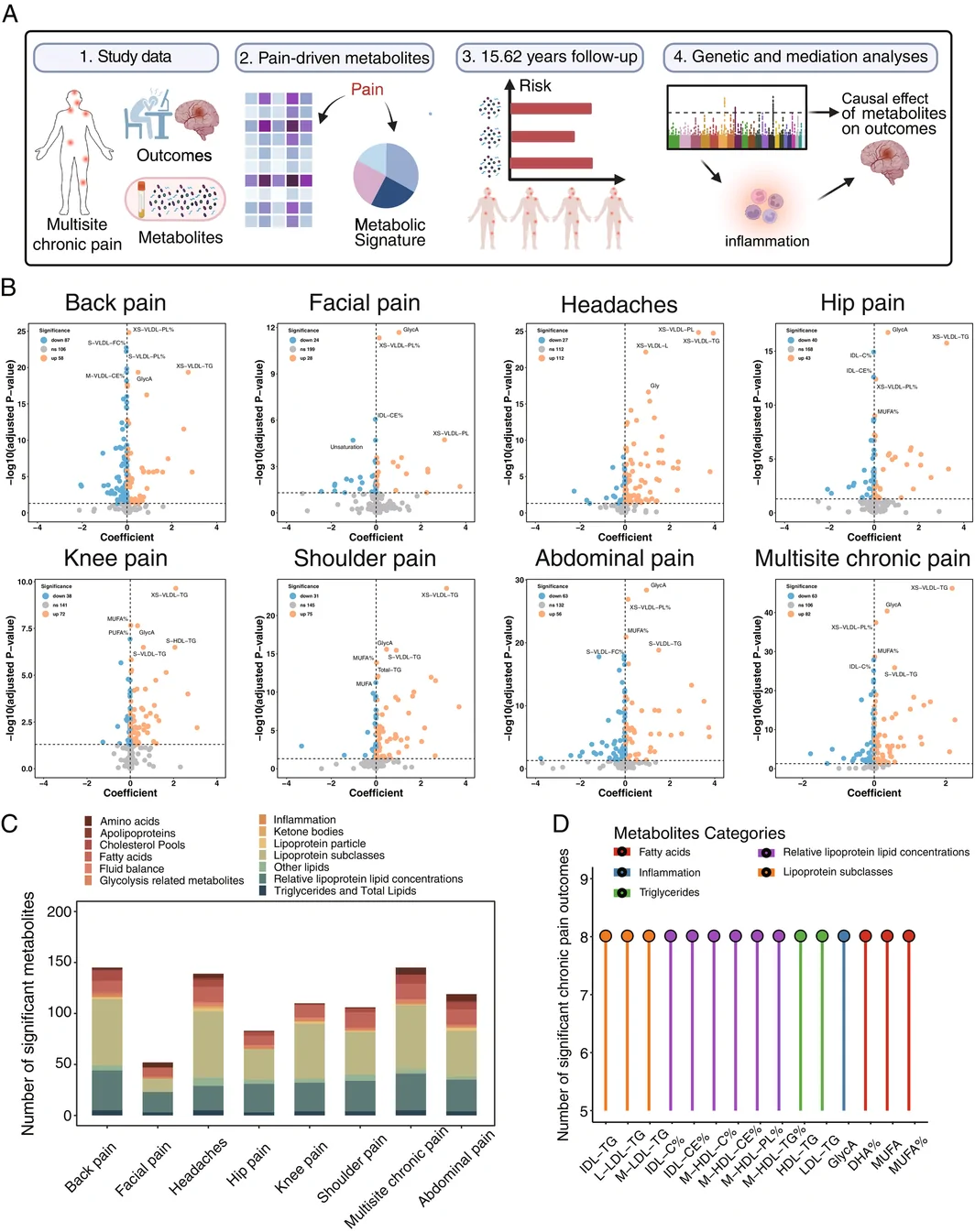
Study overview and atlas of metabolite–chronic pain association analysis results. (A) Flowchart of the study. Created with BioRender.com. (B) Association between 8 chronic pain outcomes and 251 plasma metabolites. Metabolites above the horizontal black dotted line were significantly associated with chronic pain outcomes after FDR correction (FDR‐corrected *p* < 0.05) and covariate adjustment, including age, sex, ethnicity, educational attainment, the Townsend deprivation index, smoking status, alcohol consumption, body mass index, family history of brain disorders, and prevalent hypertension and diabetes. (C) Plots show the number of each category of metabolites significantly associated with chronic pain outcomes. (D) Number of chronic pain outcomes by metabolite chapter.

**TABLE 1:** Characteristics of study participants at baseline by multisite chronic pain.

Characteristics	Overall participants	Multisite chronic pain				*p* ^a^
		0	1–3	4‐5	6‐7	
Number of participants	174, 630	97, 992	68, 957	4863	2818	—
Age, years	56.56 (8.08)	56.36 (8.13)	56.81 (8.04)	56.91 (7.71)	56.71 (7.73)	< 0.001
Sex, male, *n* (%)	94, 631 (54.19)	52, 132 (53.20)	38, 013 (55.13)	2879 (59.20)	1607 (57.03)	< 0.001
Ethnicity, White, *n* (%)	165, 892 (95)	93, 480 (95.40)	65, 363 (94.79)	4532 (93.19)	2517 (89.32)	< 0.001
Townsend index	−1.34 (3.08)	−1.43 (3.02)	−1.28 (3.11)	−0.91 (3.25)	−0.55 (3.48)	< 0.001
Body mass index, kg/m^2^	27.44 (4.78)	26.88 (4.39)	27.97 (5.02)	29.63 (5.92)	29.81 (6.03)	< 0.001
Higher education level, *n* (%)	122, 364 (70.07)	70, 337 (71.78)	47, 207 (68.46)	3093 (63.60)	1727 (61.28)	< 0.001
Hypertension, *n* (%)	100, 565 (57.59)	55, 305 (56.44)	40, 423 (58.62)	3018 (62.06)	1819 (64.55)	< 0.001
Diabetes, *n* (%)	7126 (4.08)	3454 (3.52)	3060 (4.44)	331 (6.81)	281 (9.97)	< 0.001
Family history of brain disorders, *n* (%)	74, 709 (42.78)	40, 350 (41.18)	30, 501 (44.23)	2470 (50.79)	1388 (49.25)	< 0.001
Smoke, *n* (%)						< 0.001
Current	18, 394 (10.53)	9166 (9.35)	7903 (11.46)	790 (16.25)	535 (18.99)	
Never	95, 774 (54.84)	56, 322 (57.48)	35, 858 (52)	2248 (46.23)	1346 (47.76)	
Previous	60, 462 (34.62)	32, 504 (33.17)	25, 196 (36.54)	1825 (37.53)	937 (33.25)	
Alcohol, *n* (%)						< 0.001
Current	160, 744 (92.05)	91, 104 (92.97)	63, 103 (91.51)	4216 (86.70)	2321 (82.36)	
Never	7672 (4.39)	3968 (4.05)	3164 (4.59)	327 (6.72)	213 (7.56)	
Previous	6214 (3.56)	2920 (2.98)	2690 (3.90)	320 (6.58)	284 (10.08)	
Medication use, *n* (%)						
Antidepressants	11, 805 (6.76)	4854 (4.95)	5556 (8.06)	808 (16.62)	587 (20.83)	< 0.001
Aspirin	25, 474 (14.59)	12, 970 (13.24)	10, 961 (15.90)	982 (20.19)	561 (19.91)	< 0.001
Anti‐anxiety agents	2549 (1.46)	1326 (1.35)	1081 (1.57)	91 (1.87)	51 (1.81)	< 0.001
Lipid‐lowering agents	27, 516 (15.76)	15, 559 (15.88)	10,7 41 (15.58)	792 (16.29)	424 (15.05)	0.181

^a^ *p*‐values were calculated using one‐way analysis of variance or *χ* ^*2*^ test, as appropriate. Multisite chronic pain was defined as the number of chronic pain sites across seven body regions, ranging from 0 to 7.

### 3.2 Metabolite‐Wide Association of Chronic Pain Conditions

First, we examined associations between 251 metabolites and eight chronic pain outcomes. In the full dataset, 899 associations remained significant after adjustment for covariates and FDR correction (FDR‐corrected *p* < 0.05). The multisite chronic pain and chronic back pain exhibited the largest number of associated metabolites (*n* = 145, 16.13% each), followed by chronic headache (*n* = 139, 15.46%), abdominal pain (*n* = 119, 13.24%), knee pain (*n* = 110, 12.24%), shoulder pain (*n* = 106, 11.79%), hip pain (*n* = 83, 9.23%), and facial pain (*n* = 52, 5.78%) (Figure 1B). We quantified both the number and proportion of significant metabolite associations across the eight outcomes. As depicted in Figure 1C, the majority mapped to lipoprotein subclasses and fatty acids. Notably, 15 metabolites emerged as top pain‐related signals, each associated with all chronic pain outcomes (Figure 1D). Additional details are provided in Tables S3–S10.

### 3.3 Identification of the Multisite Chronic Pain‐Related Metabolic Signature

We then used elastic net regression with the optimal λ selected via 10‐fold cross‐validation to identify a metabolic signature associated with the number of chronic pain sites. In total, 88 metabolites were selected, mainly including lipoprotein subclasses, amino acids, and fatty acids (Figure 2A,B, Table S11). The strongest positive coefficients were primarily observed for the concentration of very small VLDL particles. In contrast, triglycerides in very large HDL contributed prominently to negative coefficients (Figure 2C). The extensive associations between the selected metabolites, number of chronic pain sites, and each pain component were also observed (Figure 2A). Additionally, participants with a higher number of chronic pain sites exhibited elevated metabolic signature scores (Figure S2).

**FIGURE 2:**
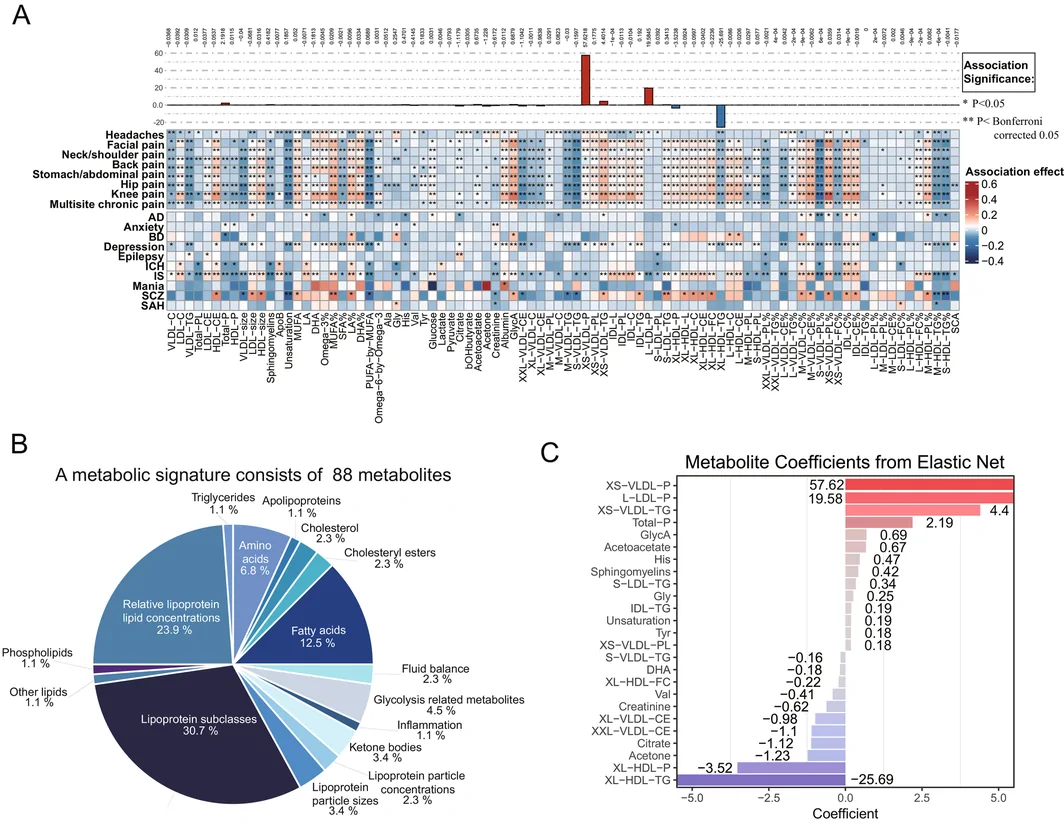
The metabolic signature associated with multisite chronic pain. (A) Associations between 88 metabolites constituting the metabolic signature, multisite chronic pain, each chronic pain site and each disorder risk. Asterisks denote the significance level of associations (*p < 0.05 and **Bonferroni‐corrected *p* < 0.05). C, cholesterol; CE, cholesteryl ester; FC, free cholesterol; L, large; M, medium; S, small; PL, phospholipid; TG, triglyceride; VLDL, very low–density lipoprotein; XL, very large; XS, very small; XXL, extremely large. (B) The composition ratio of the metabolic signature. (C) The top 25 metabolites by coefficient in metabolic signature. Multisite chronic pain was defined as the number of chronic pain sites across seven body regions, ranging from 0 to 7.

### 3.4 Associations of Chronic Pain With Brain Disorders

During a median follow‐up of 15.62 years, 38,731 participants developed a brain disorder event. The associations between multisite chronic pain and each disorder were investigated. After adjusting for potential confounders in Model 3, the number of chronic pain sites was significantly associated with the risks of depression, anxiety, and IS (Bonferroni‐corrected *p* < 0.05) (Figure 3A). Specifically, a greater number of chronic pain sites was associated with the HR of 1.13 (95% CI: 1.11–1.14) for depression, 1.10 (95% CI: 1.09–1.12) for anxiety, and 1.04 (95% CI: 1.01–1.06) for IS (Table 2). In the cross‐sectional analyses, the number of chronic pain sites was also associated with prevalent depression, anxiety, and IS (Table S12). No significant associations were observed between multisite chronic pain and the remaining seven disorders. In Kaplan–Meier analyses, log‐rank tests indicated significant differences in the cumulative incidence of depression, anxiety, and IS between the unfavorable and favorable groups defined by the multisite chronic pain (Figure 3B).

**FIGURE 3:**
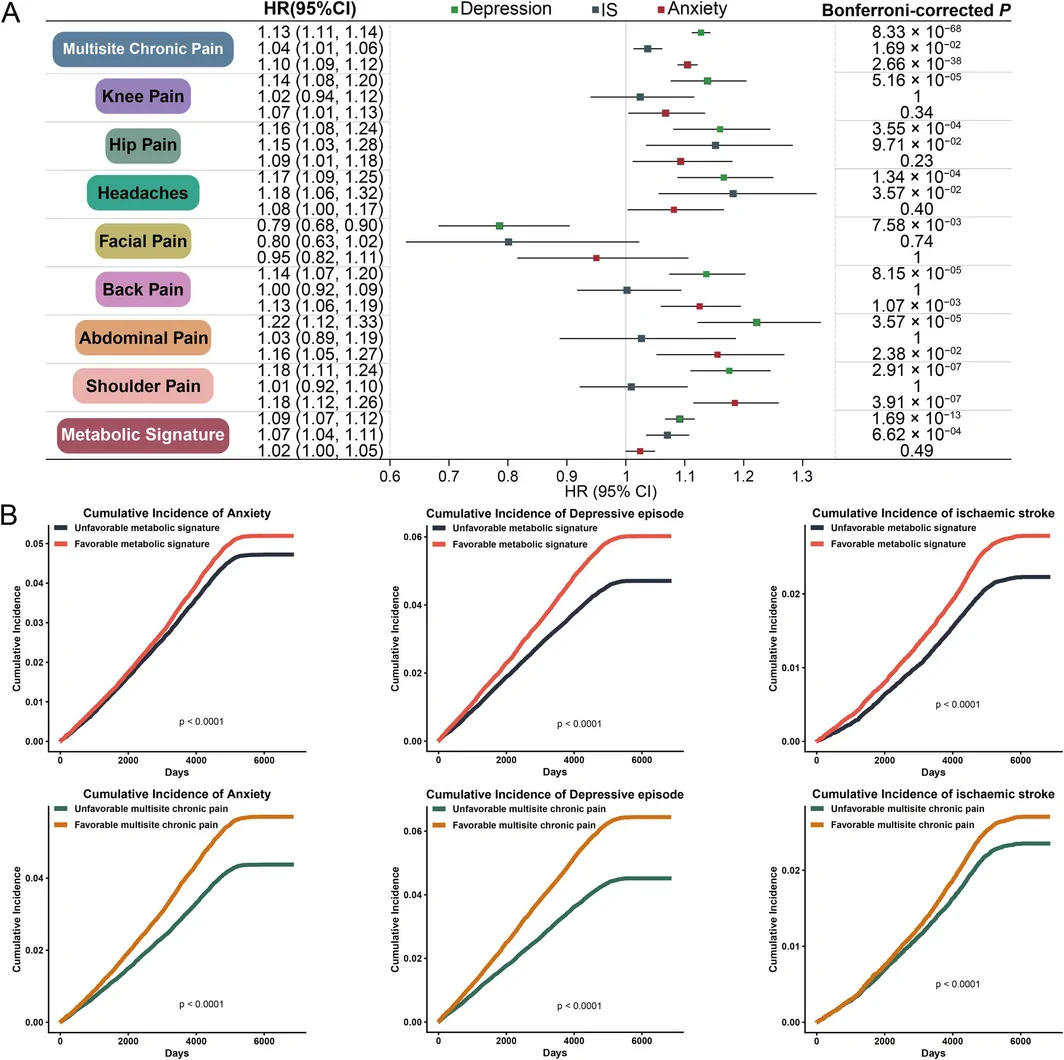
Significant associations of multisite chronic pain and metabolic signature with neurological and psychiatric disorders. (A) The panel presents the association of multisite chronic pain, each chronic pain site, and metabolic signature with depression, anxiety, and IS risk. IS, Ischemic stroke. (B) Cumulative incidence of depression, anxiety, and IS between metabolic signature groups and multisite chronic pain groups.

**TABLE 2:** Associations of multisite chronic pain and metabolic signature with depression, anxiety and ischaemic stroke risk.

Outcome	Models	Multisite chronic pain		Metabolic signature	
		HR (95% CI)^a^	*p*	HR (95% CI)^b^	*p*
Depression	Age‐ and sex‐adjusted model	1.16 (1.15, 1.18)	5.25 × 10^−115^	1.18 (1.15, 1.20)	9.06 × 10^−54^
	Multivariable‐adjusted model^c^	1.13 (1.12, 1.15)	2.06 × 10^−73^	1.11 (1.08, 1.13)	2.60 × 10^−18^
	Multivariable‐adjusted + mutual adjustment^d^	1.13 (1.11, 1.14)	8.33 × 10^−68^	1.09 (1.07, 1.12)	1.69 × 10^−13^
Ischaemic Stroke	Age‐ and sex‐adjusted model	1.07 (1.05, 1.09)	1.37 × 10^−8^	1.15 (1.11, 1.18)	2.10 × 10^−17^
	Multivariable‐adjusted model^c^	1.04 (1.02, 1.06)	5.80 × 10^−3^	1.07 (1.04, 1.11)	2.43 × 10^−4^
	Multivariable‐adjusted + mutual adjustment^d^	1.04 (1.01, 1.06)	1.69 × 10^−2^	1.07 (1.04, 1.11)	6.62 × 10^−4^
Anxiety	Age‐ and sex‐adjusted model	1.11 (1.10, 1.13)	2.47 × 10^−48^	1.06 (1.03, 1.08)	6.28 × 10^−6^
	Multivariable‐adjusted model^c^	1.11 (1.09, 1.12)	1.98 × 10^−39^	1.04 (1.01, 1.06)	0.04
	Multivariable‐adjusted + mutual adjustment^d^	1.10 (1.09, 1.12)	2.66 × 10^−38^	1.02 (1.00, 1.05)	0.485

*Note:* All *p‐*values shown are Bonferroni‐corrected *p* values.

Abbreviations: CI, confidence interval; HR, hazard ratio.

^a^ HR and 95% CI of depression, ischaemic stroke, and anxiety risk per one‐site increase in multisite chronic pain.

^b^ HR and 95% CI of depression, ischaemic stroke and anxiety risk per standard deviation (SD) increase in metabolic signatures.

^c^ Adjustment for age, sex, ethnicity, Townsend index, alcohol and tobacco use, education level, body mass index, family history of brain disorders and prevalent status of hypertension and diabetes.

^d^ Also including both multisite chronic pain and the metabolic signature simultaneously in the multivariable‐adjusted model to assess their independent associations.

Subgroup analyses consistently indicate that the number of chronic pain sites was associated with increased risks of depression, anxiety, and IS across various strata, including age (< 65 years or ≥ 65 years), sex, education level, Townsend deprivation index, BMI, and prevalent hypertension status (Tables S13–S15). In sensitivity analyses, the results remained robust after excluding participants who experienced an event within the first 3 years of follow‐up and after additional adjustment for lipid‐lowering medications, aspirin use, antidepressants, and anti‐anxiety medication (Tables S16–S21).

We also identified additional associations between seven pain sites and these three outcomes (Figure 3A). For each pain site, its association with depression risk was evaluated. Notably, participants reporting chronic abdominal pain had the highest risk of incident depression (HR = 1.22, 95% CI: 1.12–1.33). In addition, positive associations with anxiety risk were observed for all pain sites except the face. For IS, significant associations were observed for headache and chronic hip pain.

We further characterized the prevalence of neurological and psychiatric comorbidities across chronic pain groups. The proportion of participants with two or more prevalent neurological or psychiatric disorders increased with the number of chronic pain sites, and this association was supported by ordinal logistic regression (β = 0.06, Bonferroni‐corrected *p* = 7.27 × 10^−41^; Figure S3 and Table S22). Similarly, most specific pain sites were positively associated with prevalent neurological or psychiatric comorbidity (Table S22). When examining specific types of prevalent disorders, we observed that the most pronounced increases were in psychiatric disorders and in the co‐occurrence of neurological and psychiatric disorders, whereas the proportion of purely neurological disorders remained relatively stable (Figure S4).

### 3.5 Associations of Metabolic Signature With Neurological and Psychiatric Disorders

We next evaluated the association of the multisite chronic pain‐related metabolic signature with diseases. In multivariable‐adjusted analyses, there was a similar pattern of increasing hazards with increased metabolic signature for depression, anxiety, and IS (Table 2). Importantly, these findings remained significant for depression (HR: 1.09, 95% CI: 1.07–1.12) and IS (HR: 1.07, 95% CI: 1.04–1.11) after further adjustment for the number of chronic pain sites (Bonferroni‐corrected *p* < 0.05). In contrast, the association for anxiety was nominally significant (HR: 1.02, 95% CI: 1.00–1.05, *p* = 0.049) (Figure 3A, Table 2). KM analyses similarly showed divergent cumulative incidence trajectories between unfavorable and favorable signature groups (Figure 3B). In addition, the disease groups exhibited higher metabolic signature scores (Figures S5–S7). In subgroups stratified by covariates, the associations between the metabolic signature and risk of incident depression, anxiety, and IS were generally consistent across various strata (Tables S13–S15). In sensitivity analyses, the results remained robust (Tables S16–S21).

To further clarify the role of the metabolic signature, we conducted mediation analyses to determine if it partially accounts for the link between the number of chronic pain sites and incident outcomes. Our results showed that the metabolic signature significantly mediated a proportion of these associations, with 2.93% for depression (95% CI: 2.08%–3.87%), 1.23% for anxiety (95% CI: 0.41%–2.45%), and 8.36% for IS (95% CI: 2.39%–31.06%). These findings suggest that metabolic alterations may partly explain the associations between multisite chronic pain and depression, anxiety, and IS.

### 3.6 The Genetic Determinants of the Metabolic Signature and Neurological and Psychiatric Disorders

In order to better explain the association of the metabolic signature with these diseases, we used MR to analyze the genetic causality between 88 metabolites and diseases. Using the IVW method, we found 22 plasma metabolites significantly linked to depression risk and 14 to IS risk (FDR‐corrected *p* < 0.05) (Figure 4A, Tables S23–S25). Regarding anxiety, no metabolites reached the significance threshold after FDR correction, yet 7 demonstrated suggestive causal associations (*p* < 0.05). For example, higher circulating levels of docosahexaenoic acid (DHA) were significantly associated with decreased depression risk, genetically elevated apolipoprotein B (apoB) was significantly associated with higher IS risk, and genetically elevated valine was suggestively associated with anxiety risk. Sensitivity analyses yielded similar estimates (Tables S26–S28). Notably, consistent with the prospective findings (Figure 2A), 12 were associated with depression risk and 6 with IS risk. These metabolites may represent candidate biomarkers associated with disease risk (Table S29).

**FIGURE 4:**
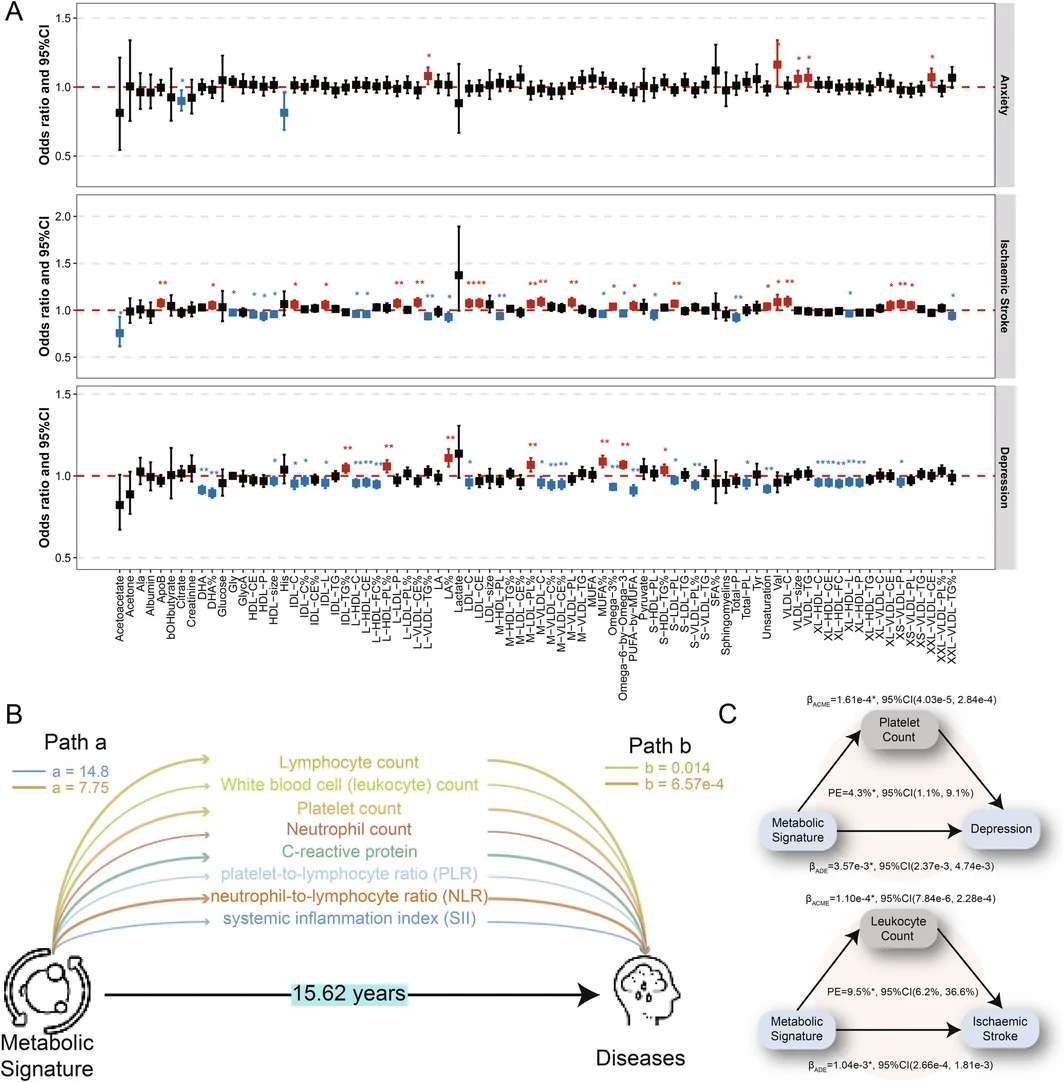
Mendelian randomization (MR) and mediation analyses results. (A) MR of associations between genetically determined metabolite with depression, anxiety, and IS (*p < 0.05 and **FDR‐corrected *p* < 0.05). (B, C) Mediation analyses showed that two of eight inflammatory markers significantly and partially mediated the prospective associations between the metabolic signature and depression and IS incidence, while controlling for numerous covariates (*FDR‐corrected *p* < 0.05). Path a: The effect of the independent variable (metabolic signature) on the mediator (inflammatory markers); Path b: The effect of the mediator (inflammatory markers) on the outcome variable (depression, anxiety, and IS).

### 3.7 Mediating Role of Inflammatory Index in the Association Between the Metabolic Signature and Neurological and Psychiatric Disorders Incidence

We investigated whether inflammatory index mediated the associations between the metabolic signature and depression, anxiety, and IS. After adjusting for covariates and FDR correction, each of the eight inflammatory indexes showed a significant association with the composite metabolic signature (Figure 4B; Figure S8; Table S30). SII showed the largest effect (*β* = 14.77, FDR‐corrected *p* = 1.35 × 10^−57^), followed by platelet count (*β* = 7.75, FDR‐corrected *p* < 0.01). In addition, most selected metabolites were significantly associated with the inflammatory indexes (Figure S8). Three inflammatory indexes were significantly associated with depression, one with IS, and four with anxiety after controlling for covariates (Figure S9; Table S31). The mediation analyses indicated that platelet count mediated the association between metabolic signature and depression, with an intermediate ratio of 4.33% (FDR‐corrected *p* < 0.05). In addition, leukocyte count significantly mediated the signature–IS association, with an intermediate ratio of 9.52% (Figure 4C). None of the eight inflammatory indexes showed significant mediation for anxiety.

## 4 Discussion

In this study, we analyzed 251 plasma metabolites in the UKB to assess associations with seven chronic pain sites and the number of chronic pain sites (multisite chronic pain). Fifteen metabolites were consistently linked to all traits. Using 88 selected metabolites, we derived a metabolic signature indicative of chronic pain. Both the signature and the number of chronic pain sites were associated with higher risks of incident depression, anxiety, and IS. Mediation analyses further indicated that the metabolic signature partially mediated the associations between multisite chronic pain and these incident outcomes. MR supported causal effects of several identified metabolites on these outcomes. Finally, platelet and leukocyte counts significantly mediated the associations between the metabolic signature and depression and IS, implicating systemic inflammation as a potential mechanism.

Our findings corroborate existing results demonstrating positive associations between chronic pain conditions and depression, anxiety, and IS. Prior studies have reported associations between chronic pain and specific neurological outcomes like stroke, Parkinson's Disease (PD), or multiple sclerosis (MS) [25, 26, 27]. Some observational studies also reported the common associations between chronic pain and psychiatric disorders, such as insomnia, anxiety, and depression [5, 28]. Few studies have examined chronic pain dimensionally rather than categorically. In this study, we used the number of chronic pain sites as a phenotype reflecting the extent of chronic pain. A higher number of chronic pain sites was associated with increased risks of depression, anxiety, and IS, consistent with a “continuum of widespreadness” hypothesis [29].

In this study, we identified a metabolic signature of 88 metabolites that was associated with multisite chronic pain and was further linked to adverse outcomes, including increased risks of depression, anxiety, and IS. Over the past decade, numerous investigations have examined metabolic alterations in specific chronic pain conditions such as migraine [12, 30], fibromyalgia [31], and osteoarthritis [32]. For example, migraine diagnosis has been associated with lower levels of apolipoprotein A1 and high‐density lipoprotein, as well as a reduced ratio of free cholesterol to total lipids [33, 34]. However, to our knowledge, no prior research has derived a metabolic signature that captures the metabolic response to chronic pain while concurrently evaluating its relationship with the long‐term risk of brain disorders. Our findings align with prior work that leverages metabolomic profiling to objectively reflect subjective health status and investigate associations with diverse disease outcomes. For instance, Tian et al. identified metabolites associated with a healthy lifestyle that were significantly related to incident dementia [35]. An observational study constructed a healthy‐lifestyle metabolic signature of 81 metabolites that was linked to a reduced risk of rheumatoid arthritis [36]. Collectively, these results support the potential of plasma metabolomics to provide indicators of chronic pain states and to inform future risk stratification and prevention strategies for brain disorders.

Blood metabolic abnormalities are known to be promising biomarkers for brain disorders. Postmortem evidence has shown reduced brain DHA levels in patients with major depressive disorder [37]. Likewise, in a variety of animal models, treatments that increase DHA content result in antidepressant‐like effects [38]. Glycine is a co‐agonist for NMDA receptors. Research suggests that glycine can improve anxiety symptoms by acting on NMDA receptors in the ventral hippocampus. Consistent with accumulating evidence from preclinical and clinical studies, we demonstrated the important role of lipids in IS, such as VLDL cholesterol, concentration of HDL particles, and apolipoprotein B [39, 40]. An increasing number of studies have examined the effects of glycine in IS, yet the findings remain controversial. Our MR analysis found glycine level was suggestively associated with a reduced risk of IS, supporting its neuroprotection effect in IS.

We provided preliminary evidence for the mechanisms underlying the link between chronic pain, metabolic signature, and incident neurological and psychiatric disorders. In the present study, we demonstrated that the chronic pain‐associated metabolic signature partially mediates the relationship of the number of chronic pain sites with depression, anxiety, and IS, respectively. Biologically, this mediating role is likely driven by a shared immunoinflammatory foundation. Recent evidence highlights a shared genetic basis linking chronic pain to systemic inflammation, such as elevated CRP levels [41]. Given the highly interactive nature of circulating metabolites and inflammatory processes, our metabolic signature likely captures this systemic immunometabolic burden. Our analyses further revealed that platelet and leukocyte (WBC) counts significantly mediated the associations of the metabolic signature with depression and IS, respectively. This aligns with independent evidence of increased peripheral inflammation in these disorders [42, 43], in which specific lipid species modulate pro‐inflammatory cytokines, driving neuroinflammation [44, 45]. For example, PUFAs exert crucial anti‐inflammatory roles [46], and high‐density lipoprotein is inversely associated with WBC count [47]. Importantly, our mutually adjusted Cox models revealed that both the number of chronic pain sites and the metabolic signature maintained independent associations with incident brain disorders. This indicates that alongside the shared immunometabolic pathways, the physical burden of multisite pain exerts largely independent detrimental effects, presumably by driving presynaptic long‐term potentiation (LTP) in the anterior cingulate cortex [48] and maladaptive structural neuroplasticity in the CNS [49]. Collectively, these findings suggest that the link between chronic pain and brain disorders is highly multifactorial, potentially involving a combination of shared systemic immunometabolic dysregulation and the distinct neurobiological consequences of chronic pain.

Nonetheless, several limitations warrant consideration. First, UK Biobank participants were predominantly of European ancestry, healthier, and socioeconomically advantaged [50], which may introduce “healthy volunteer” selection bias and limit generalizability. Nevertheless, participants need not be representative of the “target” populations when estimating relative risk factor associations, as expected theoretically [51] and empirically demonstrated for the UKB [52]. Previous studies have also shown the exposure‐outcome association to be broadly similar to that of population‐representative studies, adding confidence to our findings [50]. However, caution is still warranted when extrapolating these findings to clinical or more diverse populations. Second, metabolomic profiling was measured once at baseline. Although the metabolite measurements were extensive, they remain subject to measurement error and biological variability. Accordingly, our analyses could not capture within‐individual changes over time, and therefore could not determine whether the identified metabolites and metabolic signature reflect stable biological processes. Previous studies suggest that many circulating metabolites show reasonable reproducibility across repeated measurements [53, 54]. Nevertheless, validation in studies with repeated multi‐timepoint metabolomic measurements remains necessary. Third, chronic pain and metabolomic data were collected at baseline, which limits the evaluation of their dynamic relationships, and potential changes over time may alter the strength of the results. Fourth, the functions and mechanisms of several identified metabolites in brain disorders remain incompletely understood, which limits the interpretation of the MR findings. In addition, causal inferences from MR analyses should be interpreted with caution because potential pleiotropy and heterogeneity may bias or reduce the precision of estimated causal effects [55]. Fifth, chronic pain is a heterogeneous condition that may vary with pain tolerance, the pain‐measurement scale used, and treatment status [56]. Although the multisite chronic pain has been widely used as a proxy measure reflecting the extent of chronic pain, further studies with more detailed pain phenotyping are needed to better characterize pain severity. Furthermore, in our study, the elastic‐net regression was used to calculate the metabolic signature. While we employed ten‐fold cross‐validation to minimize overfitting, this strategy does not entirely eliminate potential bias, making it difficult to disentangle true physiological meaning from correlations driven by lifestyle, socioeconomic factors, and pain. Additionally, given the significant associations observed between chronic pain and brain disorders, we cannot completely rule out the possibility that underlying or developing neurological and psychiatric conditions might have partially influenced the baseline metabolic signature. Future studies with independent cohort validation are needed to strengthen applicability in broader settings.

## 5 Conclusion

Our study demonstrates that plasma metabolic alterations associated with chronic pain are associated with an increased risk of depression, anxiety, and IS, with inflammatory markers mediating these associations. These findings provide new insights into identifying high‐risk populations and may help inform future prevention and early intervention strategies through metabolomic profiling.

## Author Contributions

All authors read and approved the final version of the manuscript. Lin Pan, Sheng Zhong, Yinsheng Chen, and Yonggao Mou conceived the ideas and designed experiments; Wenzhuo Yang and Zijian Chen wrote the manuscript; Zijian Chen, Leyan Chen, Kaijun Sun, and Yuxin Liu carried out experiments; Lin Pan, Weijie Ding, Xinhui Wang, Hongyu Chen, and Sheng Zhong analyzed the results of the experiments. Sheng Zhong and Yinsheng Chen revised the manuscript, figures, and tables.

## Funding

This study was supported by the National Natural Science Foundation of China (grant nos. 82303310 to SZ) and Science and Technology Projects in Guangzhou (2024B03J1290 to Yinsheng Chen).

## Ethics Statement

The UK Biobank received ethical approval from the North West Multi‐Centre Research Ethics Committee (MREC approval number: 11/NW/0382).

## Consent

Written informed consent was obtained from all participants in the UK Biobank.

## Conflicts of Interest

The authors declare no conflicts of interest.

## Supporting information


**Figure S1:** Flowchart of the UK Biobank participants included and excluded in study.
**Figure S2:** The distribution of the metabolic signature between different multisite chronic pain groups.
**Figure S3:** Association between the number of chronic pain sites and prevalent neurological and psychiatric comorbidities in the UK Biobank cohort. The stacked bar chart illustrates the proportion of participants (%) with 0, 1, and ≥ 2 prevalent neurological or psychiatric disorders across different groups based on the number of chronic pain sites (0, 1, 2, 3, and ≥ 4). The sample size (n) for each pain site category is indicated below the x‐axis. Overlaid lines with data points highlight the trend in these proportions across the groups.
**Figure S4:** Proportion of specific types of prevalent neurological and psychiatric comorbidities according to the number of chronic pain sites. The stacked bar chart illustrates the proportion of participants (%) categorized by the specific composition of prevalent conditions across groups defined by the number of chronic pain sites (0, 1, 2, 3, and ≥ 4). Participants were independently classified into four groups: no neurological or psychiatric disorder, neurological disorder only, psychiatric disorder only, and both neurological and psychiatric disorders. The sample size for each chronic pain category is indicated below the x‐axis.
**Figure S5:** The distribution of the metabolic signature between anxiety and non‐anxiety groups.
**Figure S6:** The distribution of the metabolic signature between depression and non‐depression groups.
**Figure S7:** The distribution of the metabolic signature between ischaemic stroke and non‐ischaemic stroke groups.
**Figure S8:** Associations of specific inflammatory index with metabolic signature and metabolites. Models adjusted for age, sex, ethnicity, Townsend index, alcohol and tobacco use, education level, body mass index, family history, prevalent status of hypertension and diabetes, multisite chronic pain and the metabolic signature. Direction is shown by color, with red for positive associations and blue for inverse associations. Asterisks denote significance levels (*p < 0.05; **FDR‐adjusted p < 0.05).
**Figure S9:** Associations of specific inflammatory index with diseases. Models adjusted for age, sex, ethnicity, Townsend index, alcohol and tobacco use, education level, body mass index, family history, prevalent status of hypertension and diabetes, multisite chronic pain (MCP) and the metabolic signature. Direction is shown by color, with red for positive associations and blue for inverse associations. Asterisks denote significance levels (*p < 0.05; **FDR‐adjusted p < 0.05).

**Table S1:** Definitions, and measurements of pain conditions in the UK Biobank study.
**Table S2:** Metadata for NMR (Nuclear Magnetic Resonance) metabolic biomarkers.
**Table S3:** Associations of metabolites with chronic headache after covariates adjustment and FDR correction.
**Table S4:** Associations of metabolites with chronic facial pain after covariates adjustment and FDR correction.
**Table S5:** Associations of metabolites with chronic neck/shoulder pain after covariates adjustment and FDR correction.
**Table S6:** Associations of metabolites with chronic back pain after covariates adjustment and FDR correction.
**Table S7:** Associations of metabolites with chronic stomach/abdominal pain after covariates adjustment and FDR correction.
**Table S8:** Associations of metabolites with chronic hip pain after covariates adjustment and FDR correction.
**Table S9:** Associations of metabolites with chronic knee pain after covariates adjustment and FDR correction.
**Table S10:** Associations of metabolites with multisite chronic pain after covariates adjustment and FDR correction.
**Table S11:** Selected metabolites from the optimized elastic net regression model.
**Table S12:** Associations between the number of chronic pain sites and prevalent neurological or psychiatric disorders.
**Table S13:** Associations between the multisite chronic pain and metabolic signature with depression risk, stratified by age, sex, body mass index, Townsend deprivation index, education level, prevalent status of hypertension and diabetes.
**Table S14:** Associations between the multisite chronic pain and metabolic signature with ischaemic stroke risk, stratified by age, sex, body mass index, Townsend deprivation index, education level, prevalent status of hypertension and diabetes.
**Table S15:** Associations between the multisite chronic pain and metabolic signature with anxiety risk, stratified by age, sex, body mass index, Townsend deprivation index, education level, prevalent status of hypertension and diabetes.
**Table S16:** Associations of multisite chronic pain and metabolic signature with depression risk in sensitivity analysis excluding events occurred within 3 years of follow up.
**Table S17:** Associations of multisite chronic pain and metabolic signature with ischemic stroke risk in sensitivity analysis excluding events occurred within 3 years of follow up.
**Table S18:** Associations of multisite chronic pain and metabolic signature with anxiety risk in sensitivity analysis excluding events occurred within 3 years of follow up.
**Table S19:** Associations of multisite chronic pain and metabolic signature with depression risk in sensitivity analysis adjusting for anti‐depression drug.
**Table S20:** Associations of multisite chronic pain and metabolic signature with ischaemic stroke risk in sensitivity analysis adjusting for anti‐platelet and lipid‐lowering drugs.
**Table S21:** Associations of multisite chronic pain and metabolic signature with anxiety risk in sensitivity analysis adjusting for anti‐anxiety drug.
**Table S22:** Association between the number of chronic pain sites and prevalent neurological or psychiatric comorbidity categories.
**Table S23:** Mendelian randomization analyses of associations of genetically determined identified metabolite with depression.
**Table S24:** Mendelian randomization analyses of associations of genetically determined identified metabolite with ischaemic stroke.
**Table S25:** Mendelian randomization analyses of associations of genetically determined identified metabolite with anxiety.
**Table S26:** Sensitivity analyses of Mendelian randomization results between metabolites and depression.
**Table S27:** Sensitivity analyses of Mendelian randomization results between metabolites and ischaemic stroke.
**Table S28:** Sensitivity analyses of Mendelian randomization results between metabolites and anxiety.
**Table S29:** The metabolite markers identified by the combined cohort study and MR Analysis.
**Table S30:** Associations of metabolic signature and selected metabolites with specific inflammatory index after covariates adjustment and FDR correction.
**Table S31:** Associations of specific inflammatory index with risk of depression, ischaemic stroke and anxiety after covariates adjustment.

**Data S1:** Supporting Information.

## Data Availability

The data that support the findings of this study are available from UK Biobank. Restrictions apply to the availability of these data, which were used under license for this study. Data are available from https://www.ukbiobank.ac.uk with the permission of UK Biobank.
