# Supplementary material for: Plasma Metabolome Mediates the Link Between Chronic Pain and Brain Disorders: A Large‐Scale Cohort and Genetic Analysis
Source: CNS Neurosci Ther. 2026 Nov 10;32(11):e71234. doi: 10.1002/cns.71234 (PMC13752479; doi:10.1002/cns.71234)
Supplement: Supplementary file 1 [file CNS-32-e71234-s001.docx]

**Supplementary Figure 1. Flowchart of the UK Biobank participants included and excluded in study.**

**Supplementary Figure 2. The distribution of the metabolic signature between different multisite chronic pain groups.**

**Supplementary Figure 3. Association between the number of chronic pain sites and prevalent neurological and psychiatric comorbidities in the UK Biobank cohort.** The stacked bar chart illustrates the proportion of participants (%) with 0, 1, and ≥2 prevalent neurological or psychiatric disorders across different groups based on the number of chronic pain sites (0, 1, 2, 3, and ≥4). The sample size (n) for each pain site category is indicated below the x-axis. Overlaid lines with data points highlight the trend in these proportions across the groups.

**Supplementary Figure 4. Proportion of specific types of prevalent neurological and psychiatric comorbidities according to the number of chronic pain sites.** The stacked bar chart illustrates the proportion of participants (%) categorised by the specific composition of prevalent conditions across groups defined by the number of chronic pain sites (0, 1, 2, 3, and ≥4). Participants were independently classified into four groups: no neurological or psychiatric disorder, neurological disorder only, psychiatric disorder only, and both neurological and psychiatric disorders. The sample size for each chronic pain category is indicated below the x-axis.

**Supplementary Figure 5. The distribution of the metabolic signature between anxiety and non-anxiety groups.**

**Supplementary Figure 6. The distribution of the metabolic signature between depression and non-depression groups.**

**Supplementary Figure 7. The distribution of the metabolic signature between ischaemic stroke and non-ischaemic stroke groups.**

**Supplementary Figure 8. Associations of specific inflammatory index with metabolic signature and metabolites.** Models adjusted for age, sex, ethnicity, Townsend index, alcohol and tobacco use, education level, body mass index, family history, prevalent status of hypertension and diabetes, multisite chronic pain and the metabolic signature. Direction is shown by color, with red for positive associations and blue for inverse associations. Asterisks denote significance levels (*P < 0.05; **FDR-adjusted P < 0.05).

**Supplementary Figure 9. Associations of specific inflammatory index with diseases.** Models adjusted for age, sex, ethnicity, Townsend index, alcohol and tobacco use, education level, body mass index, family history, prevalent status of hypertension and diabetes, multisite chronic pain (MCP) and the metabolic signature. Direction is shown by color, with red for positive associations and blue for inverse associations. Asterisks denote significance levels (*P < 0.05; **FDR-adjusted P < 0.05).
