# Supplementary material for: Plasma Metabolome Mediates the Link Between Chronic Pain and Brain Disorders: A Large‐Scale Cohort and Genetic Analysis
Source: CNS Neurosci Ther. 2026 Nov 10;32(11):e71234. doi: 10.1002/cns.71234 (PMC13752479; doi:10.1002/cns.71234)
Supplement: Supplementary file 2 [file CNS-32-e71234-s002.docx]

**Supplementary Methods**

**Study population**

The UK Biobank (UKB) project is a large population-based prospective cohort study that enrolled over 500,000 participants during 2006–2010. Details are described elsewhere ^[1](https://paperpile.com/c/9Bwb7A/x6paf)^. These participants completed touchscreen questionnaires, underwent physical assessments, and provided biological samples. This study was conducted under UKB application number 217841.

In the present study, participants were excluded if they withdrew from UKB, had brain disorders at baseline, or lacked pain questionnaire or NMR metabolomics data. Finally, 174,630 participants were eligible for this study. A flowchart illustrating the selection criteria is provided in **Supplementary Figure 1**.

**Pain and metabolite profiling assessment**

At baseline, participants reported the types of pain they experienced in the last month via a touchscreen questionnaire. Participants could select from seven body regions (knee, hip, abdomen/stomach, back, shoulder/neck, face, head), a whole‑body pain option (general pain), or "none of the above". Multiple selections were permitted for the seven regional sites, whereas general pain allowed only a single choice. Individuals reporting pain at any site or "all over the body" were then asked whether it had persisted for more than 3 months, which was classified as chronic pain. In line with prior studies ^[2,3](https://paperpile.com/c/9Bwb7A/ZjtZd+2c7m3)^, multisite chronic pain was defined as the sum of body sites with chronic pain across the seven regional sites, ranging from 0 to 7. This measure was used as a proxy for the extent of chronic pain (**Supplementary Table 1)**.

NMR metabolomic profiling data were generated using the Nightingale Health platform ^[4](https://paperpile.com/c/9Bwb7A/h0x2C)^. Currently, datasets from study phases 1 and 2 are accessible. These phases encompassed 251 metabolic biomarkers (170 quantified in absolute concentrations and 81 reported as composite ratio indices), measured in EDTA plasma from approximately 280,000 UK Biobank participants (**Supplementary Table 2**). A high-throughput NMR-based biomarker platform was employed, with detailed protocols for profiling and quality control described online. The biomarkers include diverse metabolic pathways, including low molecular weight metabolites such as amino acids, ketone bodies, and glycolysis metabolites, as well as lipoprotein lipids, fatty acids, and fatty acid composition.

**Neurological and psychiatric disorders events**

The primary outcome was defined as a composite of first-episode neurological and psychiatric disorders, including ischemic stroke (IS), intracerebral hemorrhage (ICH), subarachnoid hemorrhage (SAH), depression, anxiety, bipolar disorder (BD), schizophrenia (SCZ), mania, Alzheimer’s disease (AD) and epilepsy. These diseases were identified using self-reports, hospital inpatient records, death registry, and "first occurrence of health outcomes" category. Primary and secondary hospital diagnoses and causes of death for outcomes were coded using International Classification of Diseases (ICD-10), supplemented by UKB algorithms.

**Assessment of inflammation index**

To examine whether inflammation mediates the association of the metabolic signature with brain disorders, we selected immune parameters implicated in distinct inflammatory pathways: absolute counts of lymphocytes, leukocytes (white blood cells), platelets, and neutrophils; C-reactive protein (CRP); the neutrophil-to-lymphocyte ratio (NLR) and platelet-to-lymphocyte ratio (PLR); and the systemic immune-inflammation index (SII). SII was computed as platelet count × neutrophil count / lymphocyte count ^[5](https://paperpile.com/c/9Bwb7A/TZDPC)^.

**Assessment of covariates**

A total of 11 covariates associated with neurological or psychiatric disorders were considered in the analyses. Sociodemographic factors comprised age, sex, ethnicity (white versus others), educational attainment (college or university degree versus other), and material deprivation (measured using the Townsend deprivation index). The Townsend deprivation index was derived from the output area corresponding to each participant’s postcode, with higher scores reflecting greater deprivation. Lifestyle and clinical factors included smoking status (never, previous, current), alcohol consumption (never, previous, current), weight status based on body mass index, family history of neurological or psychiatric disorders, and prevalent hypertension and diabetes.

**Identification of chronic pain condition-related metabolites**

Multivariable linear regression models were used to assess the associations between 251 metabolite levels and pain conditions across seven body sites, as well as the number of chronic pain sites. To control for potential confounders, these models were adjusted for all 11 aforementioned covariates. Prior to analysis, we applied log transformation and standardized all metabolites to the same scale. Significance was defined by a false discovery rate (FDR)-corrected *P* < 0.05.

To derive a metabolic signature for chronic pain, we fitted an elastic net regression, which blends lasso and ridge regression penalties to address multicollinearity, reduce overfitting, and enable feature selection ^[6](https://paperpile.com/c/9Bwb7A/iJszt)^. The multisite chronic pain was regressed on all 251 standardized metabolites, and the optimal penalty parameter λ was selected via 10-fold cross-validation using the one-standard-error rule. We then computed a total metabolic signature as the weighted sum of the selected metabolites, with weights equal to the elastic net coefficients.

**Associations of pain conditions and metabolic signature with neurological and psychiatric disorders**

In the UKB prospective cohort, we estimated HRs and 95% CIs using Cox proportional hazards models. We conducted two analyses: (1) associations of the number of chronic pain sites and each individual pain site with the brain disorders; and (2) associations of the metabolic signature with the brain disorders. Model 1 adjusted for age and sex. Model 2 additionally adjusted for all previously described covariates. Model 3 included both the chronic pain score and the metabolic signature to evaluate their independent associations. Proportional hazards were assessed via Schoenfeld residuals, with no violations observed. Multiple testing correction was applied to each model, and statistical significance was defined as a Bonferroni-corrected *P* < 0.05. For disorders meeting the significance threshold, Kaplan–Meier curves were generated to compare risk across metabolic signature groups and categories of chronic pain sites.

We also assessed the association between the number of chronic pain sites and prevalent neurological or psychiatric disorders among 146,460 participants, after excluding individuals with missing data on chronic pain, NMR metabolomics, or covariates, as well as those who developed incident brain disorders during follow-up (**Supplementary Figure 1**). First, logistic regression models were fitted to estimate the odds ratios (ORs) and 95% CIs for each prevalent disease in relation to the number of chronic pain sites, adjusting for all covariates. Additionally, we characterise the comorbidity of chronic pain with neurological and psychiatric disorders among these 146,460 participants. Participants were classified based on the number of prevalent neurological or psychiatric disorders across the 10 predefined outcomes, using ordered categories of 0, 1, or ≥2 disorders. To evaluate the specific composition of prevalent conditions, we independently categorised participants by disorder type: no neurological or psychiatric disorder, neurological disorder only, psychiatric disorder only, or both neurological and psychiatric disorders. Ordinal logistic regression was employed to examine the associations of the number of chronic pain sites and site-specific chronic pain with prevalent neuropsychiatric comorbidity, adjusting for covariates. To account for multiple comparisons across the prevalent outcomes, significance was defined by a Bonferroni-corrected *P* < 0.05.

**Subgroups and sensitivity analysis**

We assessed heterogeneity via stratified analyses by age, sex, education, BMI, and histories of hypertension and diabetes. To evaluate robustness, we performed two sensitivity analyses: (1) excluding participants who developed incident outcomes within the first 3 years of follow-up; and (2) additionally adjusting for antidepressants, anti-anxiety medications, lipid-lowering medications, and aspirin use.

**Causal inferences using MR**

We conducted MR analyses to infer causal effects of genetically predicted metabolic profiles on the risks of brain disorders. For all identified metabolites, summary statistics were sourced from a previously published study of 115,078 UKB participants of European ancestry. GWAS summary data for depression were obtained from FinnGen (Ncases = 47,696; Ncontrols = 359,290). The summary statistics for anxiety were obtained from a recent study of 41,752 European-ancestry participants ^[7](https://paperpile.com/c/9Bwb7A/qyTR8)^. The data for IS were retrieved from GWAS Catalog (https://www.ebi.ac.uk/gwas/home) (Ncases = 62,100; Ncontrols =1,234,808) ^[8](https://paperpile.com/c/9Bwb7A/shBM4)^. We selected significant SNPs (*P* < 5 × 10^−8^) and performed LD clumping (r^2^ < 0.1; 10,000 kb). The inverse-variance weighted (IVW) method was the primary estimator ^[9](https://paperpile.com/c/9Bwb7A/dZh2W)^. MR-Egger, Weighted median, simple mode and weighted mode were implemented as sensitivity approaches. Heterogeneity was evaluated with Cochran’s Q test, and horizontal pleiotropy was assessed using the MR-Egger intercept ^[10](https://paperpile.com/c/9Bwb7A/9X4GO)^. To account for multiple testing, FDR correction was applied. Associations with FDR-corrected *P* < 0.05 were considered statistically significant, and a nominal *P* < 0.05 was considered suggestive.

**Mediation analyses**

For the significant associations identified in the Cox models, we performed mediation analyses to evaluate two potential pathways. First, we implemented a standard three-variable path model to quantify the extent to which the metabolic signature mediated the associations between multisite chronic pain and incident depression, anxiety, and IS. Second, we assessed whether inflammatory markers mediated the associations between the metabolic signature and incident depression, anxiety, and IS. All analyses were performed using the mediation package in R.

**Reference**

1.	[Sudlow, C.](http://paperpile.com/b/9Bwb7A/x6paf) *[et al.](http://paperpile.com/b/9Bwb7A/x6paf)* [UK biobank: an open access resource for identifying the causes of a wide range of complex diseases of middle and old age.](http://paperpile.com/b/9Bwb7A/x6paf) *[PLoS Med.](http://paperpile.com/b/9Bwb7A/x6paf)* **[12](http://paperpile.com/b/9Bwb7A/x6paf)**[, e1001779 (2015).](http://paperpile.com/b/9Bwb7A/x6paf)

2.	[Koller, D.](http://paperpile.com/b/9Bwb7A/ZjtZd) *[et al.](http://paperpile.com/b/9Bwb7A/ZjtZd)* [Pleiotropy and genetically inferred causality linking multisite chronic pain to substance use disorders.](http://paperpile.com/b/9Bwb7A/ZjtZd) *[Mol. Psychiatry](http://paperpile.com/b/9Bwb7A/ZjtZd)* **[29](http://paperpile.com/b/9Bwb7A/ZjtZd)**[, 2021–2030 (2024).](http://paperpile.com/b/9Bwb7A/ZjtZd)

3.	[Johnston, K. J. A.](http://paperpile.com/b/9Bwb7A/2c7m3) *[et al.](http://paperpile.com/b/9Bwb7A/2c7m3)* [Genome-wide association study of multisite chronic pain in UK Biobank.](http://paperpile.com/b/9Bwb7A/2c7m3) *[PLoS Genet.](http://paperpile.com/b/9Bwb7A/2c7m3)* **[15](http://paperpile.com/b/9Bwb7A/2c7m3)**[, e1008164 (2019).](http://paperpile.com/b/9Bwb7A/2c7m3)

4.	[Würtz, P.](http://paperpile.com/b/9Bwb7A/h0x2C) *[et al.](http://paperpile.com/b/9Bwb7A/h0x2C)* [Quantitative serum nuclear magnetic resonance metabolomics in large-scale epidemiology: A primer on -omic technologies.](http://paperpile.com/b/9Bwb7A/h0x2C) *[Am. J. Epidemiol.](http://paperpile.com/b/9Bwb7A/h0x2C)* **[186](http://paperpile.com/b/9Bwb7A/h0x2C)**[, 1084–1096 (2017).](http://paperpile.com/b/9Bwb7A/h0x2C)

5.	[Hu, B.](http://paperpile.com/b/9Bwb7A/TZDPC) *[et al.](http://paperpile.com/b/9Bwb7A/TZDPC)* [Systemic immune-inflammation index predicts prognosis of patients after curative resection for hepatocellular carcinoma.](http://paperpile.com/b/9Bwb7A/TZDPC) *[Clin. Cancer Res.](http://paperpile.com/b/9Bwb7A/TZDPC)* **[20](http://paperpile.com/b/9Bwb7A/TZDPC)**[, 6212–6222 (2014).](http://paperpile.com/b/9Bwb7A/TZDPC)

6.	[Zou, H. & Hastie, T. Regularization and variable selection via the elastic net.](http://paperpile.com/b/9Bwb7A/iJszt) *[J. R. Stat. Soc. Series B Stat. Methodol.](http://paperpile.com/b/9Bwb7A/iJszt)* **[67](http://paperpile.com/b/9Bwb7A/iJszt)**[, 301–320 (2005).](http://paperpile.com/b/9Bwb7A/iJszt)

7.	[Jiang, L., Zheng, Z., Fang, H. & Yang, J. A generalized linear mixed model association tool for biobank-scale data.](http://paperpile.com/b/9Bwb7A/qyTR8) *[Nat. Genet.](http://paperpile.com/b/9Bwb7A/qyTR8)* **[53](http://paperpile.com/b/9Bwb7A/qyTR8)**[, 1616–1621 (2021).](http://paperpile.com/b/9Bwb7A/qyTR8)

8.	[Mishra, A.](http://paperpile.com/b/9Bwb7A/shBM4) *[et al.](http://paperpile.com/b/9Bwb7A/shBM4)* [Stroke genetics informs drug discovery and risk prediction across ancestries.](http://paperpile.com/b/9Bwb7A/shBM4) *[Nature](http://paperpile.com/b/9Bwb7A/shBM4)* **[611](http://paperpile.com/b/9Bwb7A/shBM4)**[, 115–123 (2022).](http://paperpile.com/b/9Bwb7A/shBM4)

9.	[Zhong, S.](http://paperpile.com/b/9Bwb7A/dZh2W) *[et al.](http://paperpile.com/b/9Bwb7A/dZh2W)* [Association between viral infections and glioma risk: a two-sample bidirectional Mendelian randomization analysis.](http://paperpile.com/b/9Bwb7A/dZh2W) *[BMC Med.](http://paperpile.com/b/9Bwb7A/dZh2W)* **[21](http://paperpile.com/b/9Bwb7A/dZh2W)**[, 487 (2023).](http://paperpile.com/b/9Bwb7A/dZh2W)

10.	[Bowden, J., Davey Smith, G. & Burgess, S. Mendelian randomization with invalid instruments: effect estimation and bias detection through Egger regression.](http://paperpile.com/b/9Bwb7A/9X4GO) *[Int. J. Epidemiol.](http://paperpile.com/b/9Bwb7A/9X4GO)* **[44](http://paperpile.com/b/9Bwb7A/9X4GO)**[, 512–525 (2015).](http://paperpile.com/b/9Bwb7A/9X4GO)
